# Comparative accuracy of artificial intelligence versus manual interpretation in detecting pulmonary hypertension across chest imaging modalities: a diagnostic test accuracy meta-analysis

**DOI:** 10.3389/frai.2025.1709489

**Published:** 2026-01-13

**Authors:** Faizan Ahmed, Faseeh Haider, Ramsha Ali, Muhammad Arham, Yusra Junaid, Allah Dad, Kinza Bakht, Maryam Abbasi, Bareera Tanveer Malik, Abdul Mateen, Najam Gohar, Rubiya Ali, Yasar Sattar, Mushood Ahmed, Mohamed Bakr, Swapnil Patel, Jesus Almendral, Fawaz Alenezi

**Affiliations:** 1 Department of Medicine, Jersey Shore University Medical Center, Hackensack Meridian Health, Neptune, NJ, United States; 2 Department of Medicine, Allama Iqbal Medical College, Lahore, Pakistan; 3 Peoples University of Medical and Health Sciences, Shaheed Benazirabad, Pakistan; 4 Sheikh Zayed Medical College, Rahim Yar Khan, Pakistan; 5 Dow University of Health Sciences, Karachi, Pakistan; 6 Jinnah Sindh Medical College, Karachi, Pakistan; 7 Shaikh Khalifa Bin Zayed Al Nahyan Medical and Dental College, Lahore, Pakistan; 8 Karachi Medical and Dental College, Karachi, Pakistan; 9 Ameer-ud-Din Medical College, Lahore, Pakistan; 10 Memorial Healthcare System, Houston, TX, United States; 11 Department of Interventional Cardiology, Tidal Health, Seaford, DE, United States; 12 Rawalpindi Medical University, Rawalpindi, Pakistan; 13 Division of Cardiology, Department of Medicine, Duke University School of Medicine, Durham, NC, United States

**Keywords:** artificial intelligence, chest imaging, diagnostic accuracy, meta-analysis, pulmonary hypertension

## Abstract

**Introduction:**

Pulmonary hypertension (PH) has an incidence of approximately 6 cases per million adults, with a global prevalence ranging from 49 to 55 cases per million adults. Recent advancements in artificial intelligence (AI) have demonstrated promising improvements in the diagnostic accuracy of imaging for PH, achieving an area under the curve (AUC) of 0.94, compared to seasoned professionals.

**Research objective:**

To systematically synthesize available evidence on the comparative accuracy of AI versus manual interpretation in detecting PH across various chest imaging modalities, i.e., chest X-ray, echocardiography, CT scan and cardiac MRI.

**Methods:**

Following PRISMA guidelines, a comprehensive search was conducted across five databases—PubMed, Embase, ScienceDirect, Scopus, and the Cochrane Library—from inception through March 2025. Statistical analysis was performed using R (version 2024.12.1 + 563) with 2 × 2 contingency data. Sensitivity, specificity, and diagnostic odds ratio (DOR) were pooled using a bivariate random-effects model (reitsma() from the mada package), while the AUC were meta-analyzed using logit-transformed values via the metagen() function from the meta package.

**Results:**

This meta-analysis of 12 studies, encompassing 7,459 patients, demonstrated a statistically significant improvement in diagnostic accuracy of PH with AI integration, evidenced by a logit mean difference in AUC of 0.43 (95% CI: 0.23–0.64; *p* < 0.0001) and low heterogeneity (*I*^2^ = 21.0%, *τ*^2^ < 0.0001, *p* = 0.2090), which was consolidated by pooled AUC of 0.934 on bivariate model. Pooled sensitivity and specificity for AI models were 0.83 (95% CI: 0.73–0.90) and 0.91 (95% CI: 0.86–0.95), respectively, with substantial heterogeneity for sensitivity (*I*^2^ = 83.8%, *τ*^2^ = 0.4934, *p* < 0.0001) and moderate for specificity (*I*^2^ = 41.5%, *τ*^2^ = 0.1015, *p* = 0.1146); the diagnostic odds ratio was 54.26 (95% CI: 22.50–130.87) with substantial heterogeneity (*I*^2^ = 70.7%, *τ*^2^ = 0.8451, *p* = 0.0023). Sensitivity analysis showed stable estimates and did not reduce heterogeneity across outcomes.

**Conclusion:**

AI-integrated imaging significantly enhances diagnostic accuracy for pulmonary hypertension, with higher sensitivity (0.83) and specificity (0.91) compared to manual interpretation across chest imaging modalities. However, further high-quality trials with externally validated cohorts may be needed to confirm these findings and reduce variability among AI models across diverse clinical settings.

## Introduction

Pulmonary Hypertension (PH) is a progressive disease characterized by mean pulmonary artery pressure >20 mm Hg on the Right Heart Catheterization (Maron, 2023). It has multiple etiologies and clinical presentations leading to significant morbidity and mortality (Manek and Bhardwaj, 2025). Recent data estimate a 1-year mortality rate of approximately 8%, which escalates to nearly 24% over 3 years, highlighting the progressive nature of the disease (Chang et al., 2022). Diagnostic chest imaging modalities such as Chest X-ray (CXR), CT scans or Echocardiography play an integral role in diagnosis of Pulmonary Hypertension. However, manual interpretation of these imaging modalities is prone to diagnostic errors and inter-observer variability, potentially contributing to missed or delayed diagnoses (Sharma et al., 2021; Brady et al., 2012). This delayed diagnosis is significantly associated with poor outcomes and increased health costs (Kubota et al., 2024). A study by Kubota et al. (2024) reported that patients who had Pulmonary Hypertension diagnosed within 3 months had significantly better survival outcomes than the ones who had it diagnosed after 3 months. These factors have led us to look for novel methods for interpretation for chest imaging that are more accurate and efficient (Brady et al., 2012).

Recent advancements in Artificial Intelligence (AI) have been showing promising results in this niche (Anderson et al., 2024; Jia et al., 2022; Zhang et al., 2018). A comprehensive study by Anderson et al. (2024) showed excellent Area Under Curve (AUC) of 0.976 in detecting CXR abnormalities. Furthermore, non-radiologist aided with AI performed equally well compared to radiologists in interpreting CXRs. Similarly, Jia et al. (2022) reported the pooled AUC of AI Algorithm models for distinguishing COVID-19 from other pneumonias on chest imaging (such as CXR, CT scan and Lung Ultrasounds) to be 0.96, signifying its excellent potential for future diagnostic interpretation tool. The results for AI-driven Echocardiography interpretation were no different, showing up to 0.87 as a value for AUC (Zhang et al., 2018). Moreover, integrating AI models is predicted to reduce healthcare costs and time enormously in coming years (Khanna et al., 2022).

There are multiple studies comparing the accuracy and efficiency of manual interpretation by physicians to AI; however, a comprehensive meta-analysis remains a gap in research. This Diagnostic Test Accuracy Meta-analysis aims to bridge this gap by systematically addressing the comparison of AI interpretation of chest imaging to the traditional methods in detecting Pulmonary Hypertension and evaluating its severity. We hypothesize that AI-Algorithms based interpretation of chest imaging can significantly outweigh traditional interpretation methods for Pulmonary Hypertension therefore, revolutionizing the diagnostic accuracy of PH in clinical practice.

## Methodology

### Protocol

This meta-analysis was conducted in accordance with the Preferred Reporting Items for Systematic Reviews and Meta-Analyses of Diagnostic Test Accuracy Studies (PRISMA-DTA) guidelines (McInnes et al., 2018).

### Data sources and search strategy

A systematic literature search was conducted across five electronic databases: PubMed, Embase, ScienceDirect, Scopus, and the Cochrane Library, from inception until April, 2025. The search strategy utilized both MeSH terms and free-text keywords, combined using Boolean operators (“AND,” “OR”), and tailored for each database. The detailed search strategy is shown in Supplementary Table S1.

All identified records were imported into Rayyan software for de-duplication and screening. Two reviewers independently screened the titles and abstracts, followed by a full-text review of potentially eligible studies. Discrepancies were resolved by discussion or adjudication by a third reviewer. We also performed backward snowballing by reviewing the reference lists of included studies to identify additional relevant publications, aided by the literature mapping tool Litmaps.

### Eligibility criteria

We included studies involving human participants of any age diagnosed with any type of pulmonary hypertension (PH), in which chest imaging modalities—such as chest X-ray (CXR), echocardiography, computed tomography (CT), magnetic resonance imaging (MRI), or right heart catheterization (RHC)—were evaluated for diagnostic purposes. Eligible studies were required to compare artificial intelligence (AI)-based interpretation of chest imaging with conventional clinician-based interpretation for the diagnosis of PH. The primary outcome was the area under the receiver operating characteristic curve (AUC), used to assess diagnostic performance. Secondary outcomes included sensitivity, specificity, and diagnostic odds ratio (DOR).

We excluded studies involving non-human subjects, case reports, case series, cross-sectional studies, editorials, review articles, commentaries, and conference abstracts. Studies lacking full-text availability or presenting incomplete diagnostic data were also excluded.

### Data extraction

Two independent reviewers conducted data extraction using a standardized form developed in Microsoft Excel. Discrepancies were resolved by discussion with a third independent reviewer. Extracted data included: first author, year of study publication, country of study, study design, AI algorithm used (including model characteristics across internal and external validation cohorts), reference standard (i.e., traditional clinician interpretation), primary and secondary outcomes, sample size, number of images analyzed, and patient comorbidities.

For studies that reported binary classification outcomes, we extracted data to construct 2 × 2 contingency tables (true positives, TP; false positives, FP; true negatives, TN; false negatives, FN) for pooled diagnostic analysis. Subgroup analyses were pre-planned based on the imaging modality used (CXR, CT, MRI, echocardiography, or RHC) to explore heterogeneity in diagnostic performance. When essential data were missing, we contacted the corresponding authors via email. If no response was received within 2 weeks, a follow-up email was sent. Studies were excluded if no reply was received within 4 weeks of the initial contact.

### Quality assessment

Risk of bias and concerns regarding applicability were assessed using the Quality Assessment of Diagnostic Accuracy Studies 2 (QUADAS-2) tool, which evaluates four domains: “patient selection,” “index test,” “reference standard,” and “flow and timing.” Each domain was assessed for risk of bias and applicability concerns using the predefined criteria specified in the QUADAS-2 manual, and categorized as low, high, or unclear risk.

Two independent reviewers conducted the assessments, and discrepancies were resolved through discussion with a third reviewer. Summary judgments for each domain across all studies are presented as traffic light plots and overall risk-of-bias graphs (Supplementary Figure S1).

### Statistical analysis

All statistical analyses were performed using R (Version 2024.12.1 + 563). Logit AUC conversion was performed before pooling AUC using the metagen function in R. Because the AUC is a bounded proportion with a skewed sampling distribution, we applied a logit transformation to stabilize variance and improve normality prior to pooling (Shim et al., 2019). Meta-analysis was performed using inverse-variance weighting of logit-transformed AUC values, and results were back-transformed to the original AUC scale for easy readers’ interpretation. The general inverse variance method with restricted maximum likelihood (REML) model estimated between-study heterogeneity (*τ*^2^). Diagnostic accuracy was modeled using a bivariate random-effects meta-analysis based on the Reitsma model, implemented via the reitsma() function from the *mada* package in R. This approach simultaneously models logit-transformed sensitivity and specificity while accounting for the correlation between them, yielding a summary receiver operating characteristic (SROC) curve with associated 95% confidence and prediction regions (Reitsma et al., 2005). Due to the inherent structure of bivariate modeling, direct pooled estimates for sensitivity and specificity are not returned in the forest plots (Shim et al., 2019). To enable reporting of pooled diagnostic metrics and facilitate forest plot visualization, we additionally performed univariate random-effects meta-analyses. The metaprop() function from the meta package was used to estimate pooled sensitivity and specificity separately, while madauni() from the mada package was applied to estimate the pooled diagnostic odds ratio (DOR). Forest plots for each measure were generated using the forest() function. While these univariate results do not account for the sensitivity-specificity covariance, they serve as a practical summary of central tendencies across studies and support the illustrated interpretation.

Influence analysis was conducted using the metainf() function (*meta* package) to assess the impact of individual studies on pooled estimates. Funnel plots were generated using funnel(), and Egger’s test was applied via metabias() to assess small-study effects. Subgroup analysis and meta-regression were planned *a priori* based on several study-level characteristics, including imaging modality (eg, chest X-ray, CT, echocardiography), attenuation vs. diagnostic endpoints, convolutional neural network (CNN) architecture, and study design.

## Results

### Summary of study selection and eligibility process

**Figure 1:**
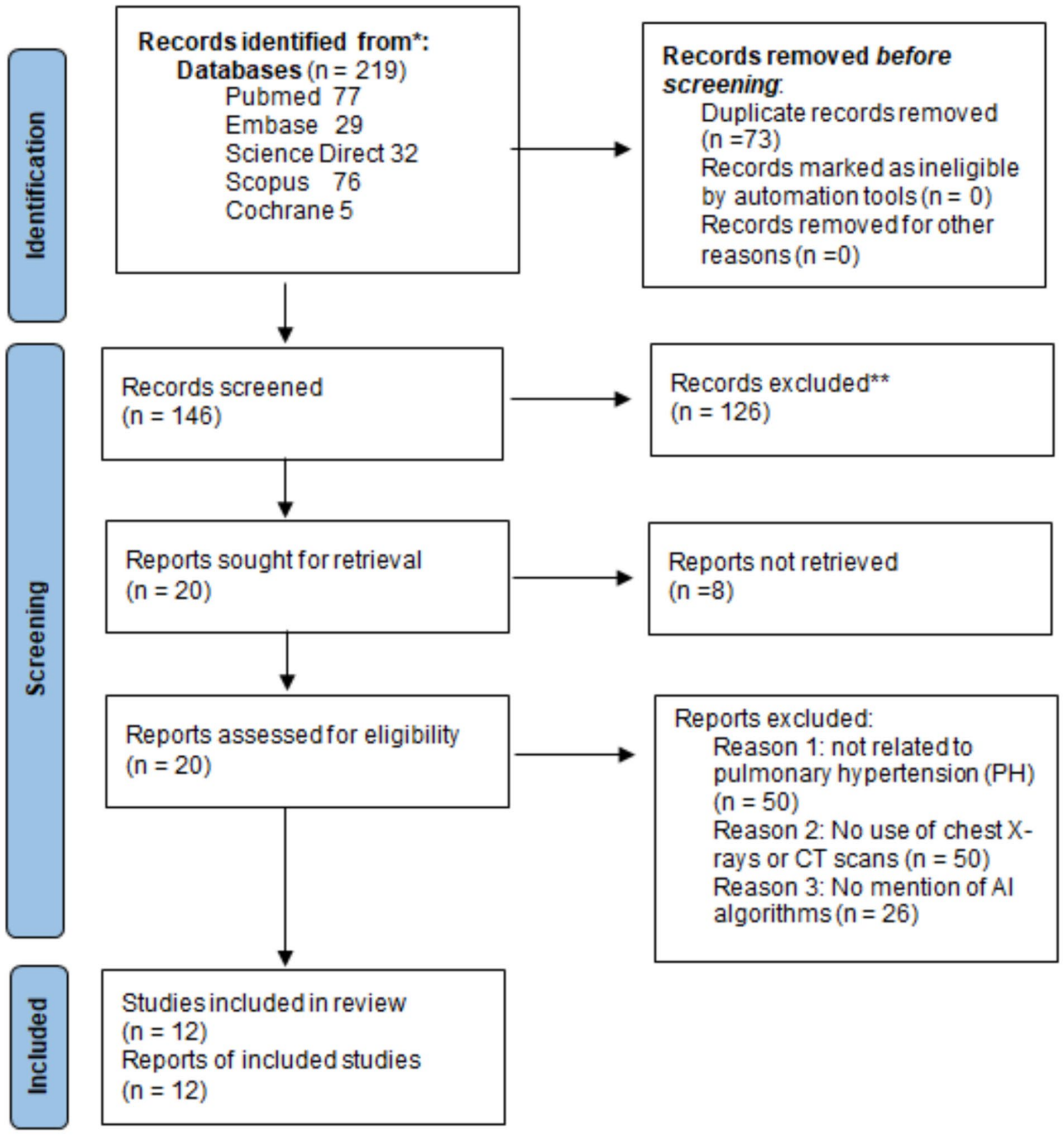
PRISMA flow chart of the included studies.

A total of 219 records were retrieved through a comprehensive search across PubMed, Embase, ScienceDirect, Scopus, and the Cochrane Library. After removing 73 duplicates, 146 records remained for title and abstract screening. Of these, 158 were excluded based on predefined eligibility criteria. Twenty full-text articles were assessed for eligibility in Rayyan, and 8 were excluded due to a lack of diagnostic AUC data, irrelevance to AI-based imaging, or unsuitable study design. An additional manual search of reference lists did not yield further eligible studies. Ultimately, 12 studies met the inclusion criteria and were included in the meta-analysis. The study selection process is detailed in the PRISMA flow diagram (Figure 1).

### Study characteristics

A total of 7,459 patients were represented across the 12 included studies, all of which evaluated AI-assisted interpretation across multiple imaging modalities. These comprised chest X-ray (CXR) (7 studies) (Imai et al., 2024; Kusunose et al., 2022; Han et al., 2024; Shimbo et al., 2024; Zou et al., 2020; Li et al., 2024; Kusunose et al., 2020), computed tomography (CT) (2 studies) (Jimenez-Del-Toro et al., 2020; Charters et al., 2022), echocardiography (2 studies) (Liao et al., 2023; Leha et al., 2019), and cardiac magnetic resonance imaging (CMR) (1 study) (Swift et al., 2021). Study characteristics and modality-specific details are summarized in Tables 1, 2, respectively.

**Table 1:** Baseline characteristics.

Study First author name (year)	Study design	Total no. of patients (*n*)	Total no. of images (*n*)	AI-algorithms group				Traditional methods group		Mean age (years/months)			Male—*n* (%)*			Congenital heart disease		Connective tissue disease	
				Internal test data		External test data													
				Total no. of patients (*n*)	Total no. of images (*n*)	Total no. of patients (*n*)	Total no. of images (*n*)	Total no. of patients (*n*)	Total no. of images (*n*)	PH group	Non-PH group	Total	PH group	Non-PH group	Total	PH group	Non-PH group	PH group	Non-PH group
Leha et al. (2019)	Retrospective cohort study	90	NA	68	NA	NA	NA	22	NA	68 ± 14 years	54 ± 19 years	61 ± 16.5 years	32 (35.5)	8 (8.8)	40 (44.4)	NA	NA	NA	NA
Zou et al. (2020)	Retrospective study	762	762	721	80	41	41	357	357	NA	NA	59.9 years	NA	NA	NA	NA	NA	NA	NA
Jimenez-Del-Toro et al. (2020)	Retrospective observational study	75	85	27	37	NA	NA	48	48	62.3 ± 15 years	65 ± 16 years	63.65 ± 15.5 years	28 (37.3)	14 (18.6)	42 (56)	NA	NA	NA	NA
Kusunose et al. (2020)	Retrospective cohort study	900	NA	90	90	55	55	900	NA	66 ± 14 years	68 ± 12 years	65.5 ± 13 years	233 (25.8)	278 (30.8)	511 (56.8)	NA	NA	NA	NA
Swift et al. (2021)	Retrospective observational study	220	NA	150	NA	NA	NA	70	NA	64 years	61 years	62.5 years	NA	NA	NA	10 (4.5)	NA	58 (26.3)	NA
Kusunose et al. (2022)	Reterospective cohort study	142	NA	NA	NA	NA	NA	NA	NA	60 ± 14 years	57 ± 13 years	58 ± 13 years	9 (6.3)	8 (5.6)	17 (12)	NA	NA	NA	NA
Charters et al. (2022)	Retrospective analysis of a prospective database	202	NA	102	NA	NA	NA	250	NA	NA	NA	NA	NA	NA	NA	NA	NA	NA	NA
Han et al. (2024)	Retrospective study	3,256	3,255	NA	330	NA	NA	NA	330	NA	NA	NA	NA	NA	1764 (54)	142 (4.3)	1,174 (36.05)	NA	NA
Liao et al. (2023)	Reterospective study	346	NA	275	NA	NA	NA	71	NA	41 ± 15 years	40 ± 15 years	40.5 ± 15 years	NA	NA	NA	NA	NA	33 (9.5)	8 (2.3)
Li et al. (2024)	Retrospective study	831	NA	166	166	50	50	NA	NA	6 ± 1.4 months	6 ± 1.2 months	6 ± 1.3 months	NA	NA	330 (49.5)	161 (19.3)	670 (80.6)	NA	NA
Shimbo et al. (2024)	Prospective cohort study	230	46	NA	NA	NA	NA	NA	NA	65.6 years	58.3 years	61.95 years	10 (16.7)	14 (8.2)	24 (24.9)	NA	NA	60 (26.08)	169 (73.4)
Imai et al. (2024)	Retrospective study	405	519	145	259	NA	NA	260	260	51.9 ± 16.1 years	62.5 ± 9.6 years	57.2 ± 12.85	34 (8.3)	131 (32.3)	165 (40.7)	17 (4.1)	NA	50 (12.3)	NA

*All the percentages are with respect to total number of patients.

AI, artificial intelligence; PH, pulmonary hypertension; *n* (%), number (percentage).

Diagnosis categories include congenital heart disease and connective tissue disease (e.g., systemic sclerosis, lupus, rheumatoid arthritis). “PH group” and “non-PH group” refer to patients with and without pulmonary hypertension, respectively.

**Table 2:** Baseline characteristics of patients by modality.

Modality	No. of studies	Total patients
Chest X-ray	7	6,526
CT scan	2	277
Echocardiography	2	436
Cardiac magnetic resonance imaging	1	220

### Quality assessment

The risk of bias was assessed using the QUADAS-2 tool, which covers four domains: patient selection, index test, reference standard, and flow and timing. It demonstrated generally acceptable methodological quality. Eight studies were judged to have low risk of bias, while four studies had either unclear or high risk, predominantly due to non-consecutive patient sampling, retrospective design, or insufficient reporting of imaging-to-reference standard timing. Applicability concerns were low overall; however, two studies used tertiary-center, highly selected patient populations, potentially limiting external validity. The risk of bias assessment is shown in Supplementary Figure S1. Importantly, inclusion of these higher-bias studies did not materially change pooled estimates as depicted in sensitivity analysis and funnel plots in results section below, though they tended to report slightly higher diagnostic accuracy, suggesting possible spectrum or selection bias.

### Logit MD AUC difference between AI and conventional methods

**Figure 2:**
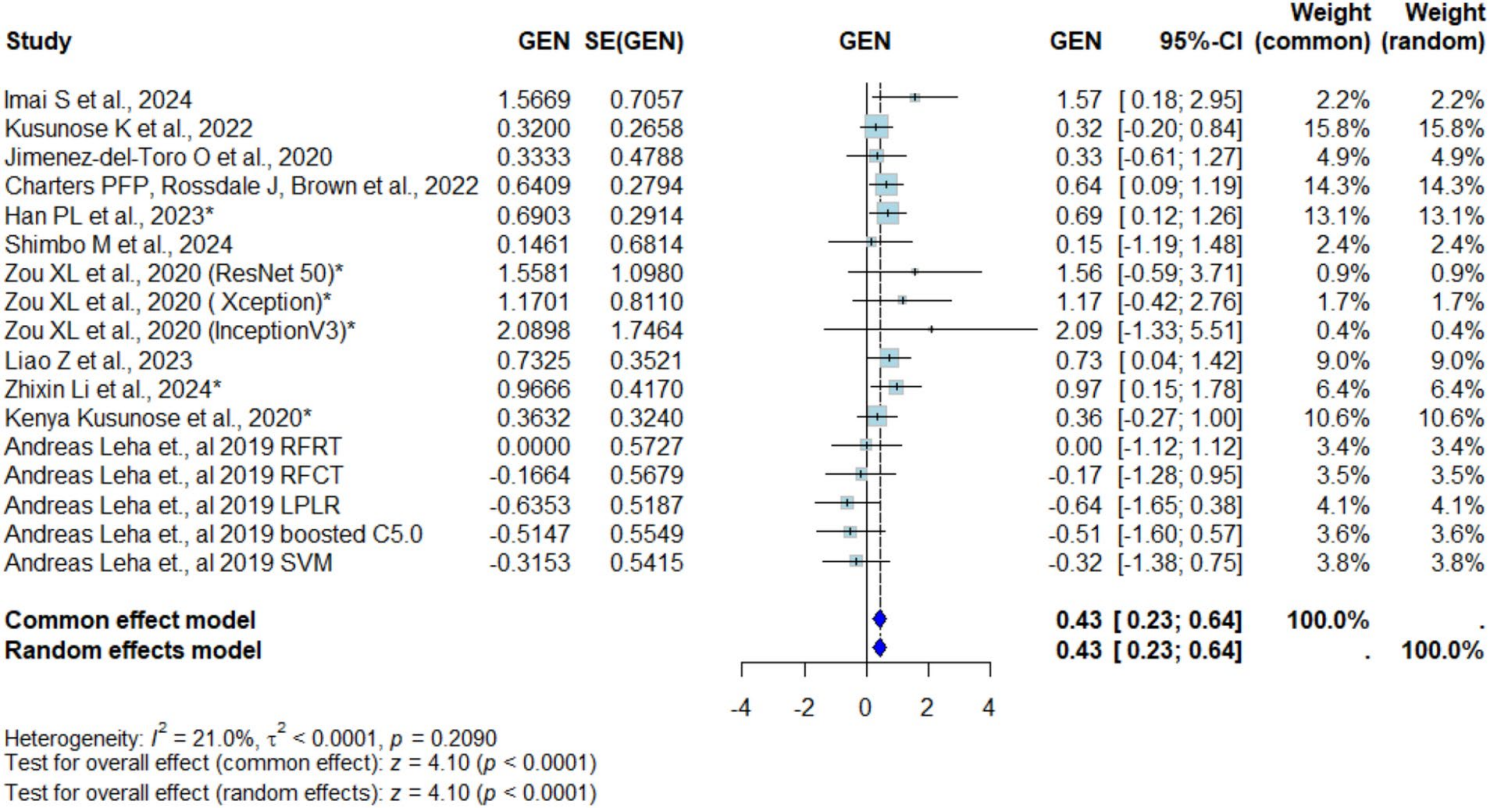
Forest plot of logit-transformed mean difference in AUC.

**Figure 3:**
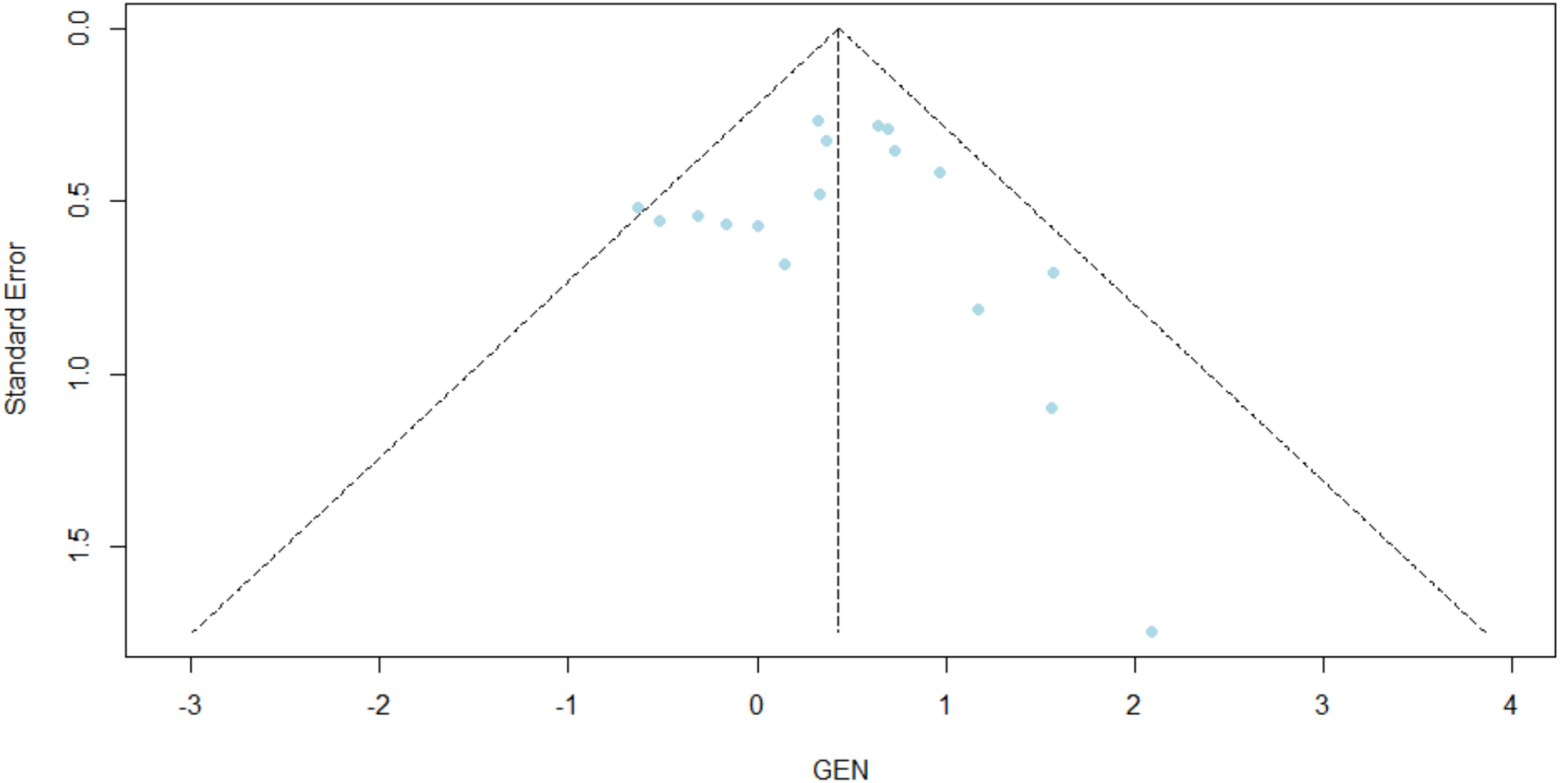
Funnel plot of logit-transformed mean difference in AUC.

Across all included imaging modalities, AI-assisted diagnostic approaches demonstrated significantly higher accuracy compared to conventional interpretation. The logit mean difference (MD) in AUC was 0.43 (95% CI: 0.23–0.64; *p* < 0.0001), indicating a statistically meaningful improvement with AI integration (Figure 2). Heterogeneity was low (*I*^2^ = 21.0%, *τ*^2^ < 0.0001, *p* = 0.2090). Egger’s test for funnel plot asymmetry revealed no evidence of small-study effects (*t* = 0.02, df = 15, *p* = 0.986), with a bias estimate of 0.0126 (SE = 0.7108) and substantial between-study heterogeneity (*τ*^2^ = 1.35) (Figure 3). Sensitivity analysis showed that excluding Leha et al. (2019) (LPLR) reduced heterogeneity substantially to 5.3%, yielding a logit-transformed AUC of 0.48 (95% CI: 0.27–0.69; *p* < 0.0001), as illustrated in Supplementary Figure S2. No evidence of small-study publication bias was detected (Egger’s test *p* = 0.986).

**Figure 4:**
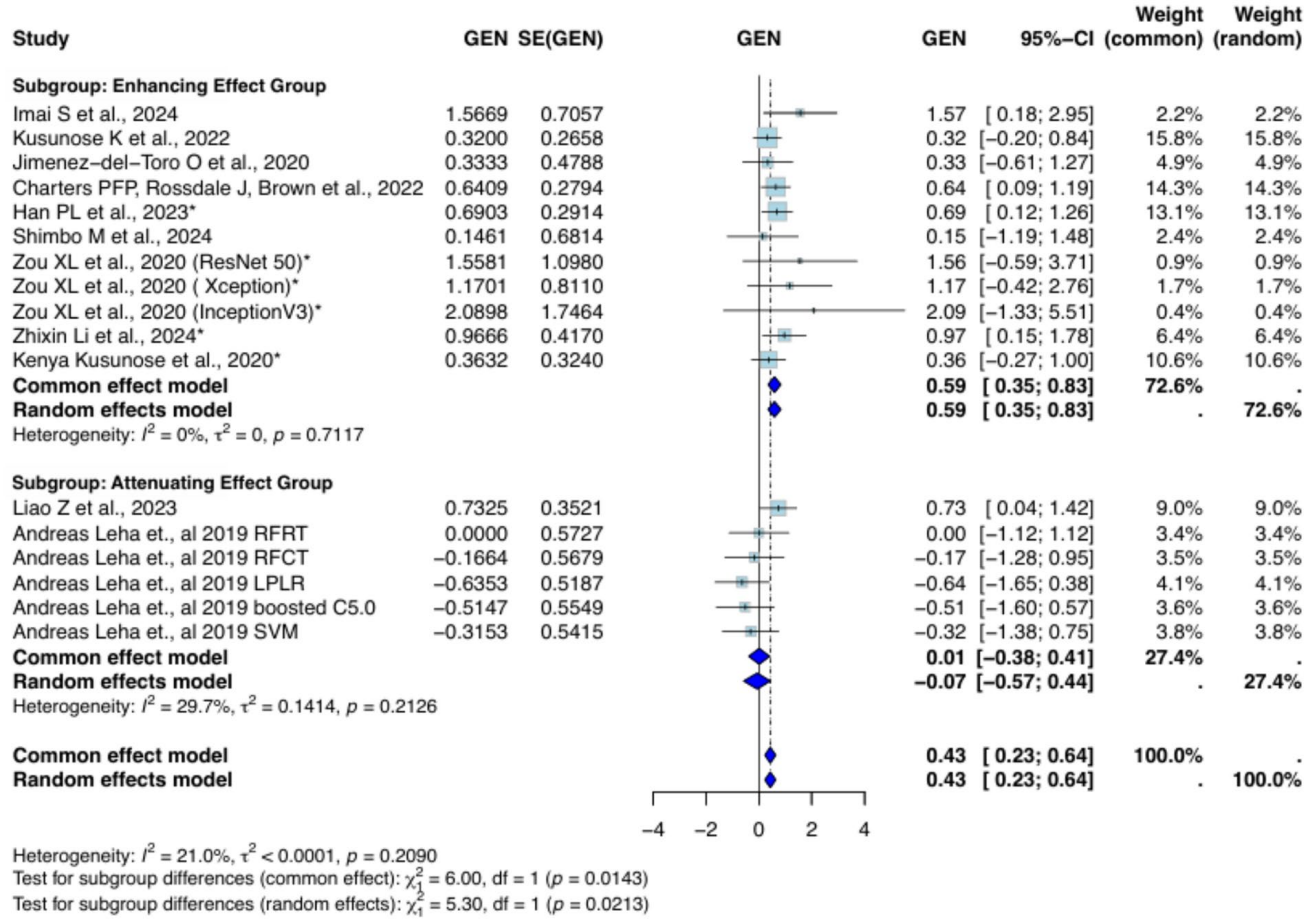
Forest plot of logit-transformed mean difference in AUC: subgroup analysis based on AUC effect enhancement.

Subgroup analysis comparing chest radiography with other imaging modalities (CT and echocardiography) demonstrated no statistically significant differences (*χ*^2^ = 3.46, df = 1, *p* = 0.063). A more granular, three-way comparison (X-ray vs. CT vs. echocardiography) similarly showed no significant subgroup effect (*χ*^2^ = 5.32, df = 2, *p* = 0.070) (Supplementary Figures S3, S4). In contrast, the analysis based on AUC effect direction revealed a significant subgroup difference (*χ*^2^ = 5.30, df = 1, *p* = 0.0213), with the enhancing-effect group demonstrating a pooled logit AUC of 0.59 (95% CI: 0.35–0.83), and no observed heterogeneity (*I*^2^ = 0%, *τ*^2^ = 0, *p* = 0.7117) (Figure 4).

### Diagnostic performance and statistical analysis

**Figure 5:**
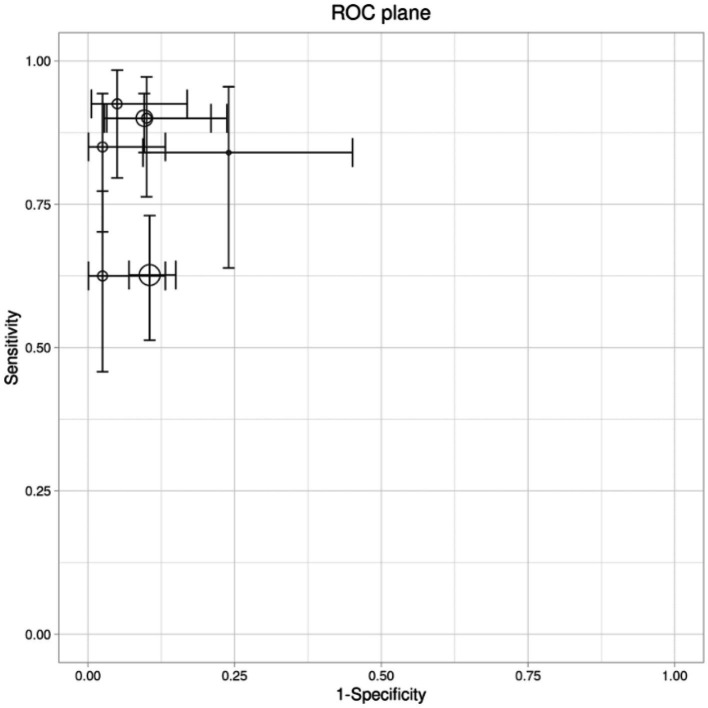
Bivariate model: ROC (receiver operating curve) plane.

A bivariate random-effects meta-analysis using the Reitsma model revealed the pooled sensitivity of 0.824 (95% CI, 0.713–0.899) and the pooled false positive rate (FPR) of 0.097 (95% CI, 0.062–0.149) for AI-based diagnostic models. The Area Under the Curve (AUC) was 0.934, with a partial AUC of 0.81, indicating strong diagnostic performance of AI-assisted methods. Heterogeneity analysis showed minimal variation in sensitivity (*I*^2^ = 27.2%) and more substantial variation in false-positive rates (FPR) (*I*^2^ = 73–82.3%). The Summary Receiver Operating Characteristic (SROC) curve for sensitivity versus 1-specificity demonstrated the overall diagnostic accuracy of AI-based models across studies (Figure 5).

The summary estimates of the bivariate model are presented in Table 3.

**Table 3:** Bivariate model: summary estimates.

Performance metrics	Estimate	95% Lower CI	95% Upper CI
Sensitivity	0.83	0.73	0.90
Specificity	0.91	0.87	0.94
DOR	49.43	23.58	103.64
LR+	9.23	6.40	13.30
LR-	0.19	0.11	0.31
FPR	0.09	0.06	0.13

DOR, diagnostic odds ratio; LR, likelihood ratio; FPR, false positive rate.

Univariate meta-analysis reinforced the bivariate model findings, reporting a pooled sensitivity of 0.83 (95% CI: 0.73–0.90) with substantial heterogeneity (*I*^2^ = 83.8%, *τ*^2^ = 0.4934, *p* < 0.0001), as shown in Supplementary Figure S5. The pooled specificity was 0.91 (95% CI: 0.86–0.95), with moderate heterogeneity (*I*^2^ = 41.5%, *τ*^2^ = 0.1015, *p* = 0.1146), as illustrated in Supplementary Figure S6. The random-effects model for diagnostic odds ratio (DOR) yielded a pooled estimate of 54.26 (95% CI: 22.50–130.87), with substantial heterogeneity (*I*^2^ = 70.7%, *τ*^2^ = 0.8451, *p* = 0.0023), as depicted in Supplementary Figure S7.

Leave-one-out analyses across sensitivity and DOR reached their lowest estimates of 74.1 and 27.3% respectively, upon exclusion of Han et al. (2024) (Supplementary Figures S8, S9). Egger’s test indicated no evidence of small-study effects for sensitivity (*t* = 1.42, df = 5, *p* = 0.214; intercept = 0.10, 95% CI: −2.18 to 2.39) or specificity (*t* = 1.23, df = 5, *p* = 0.272; intercept = 1.75, 95% CI: 0.75–2.74), whereas evidence of small-study effects was detected for DOR (*t* = 2.92, df = 5, *p* = 0.033; intercept = 1.85, 95% CI: 0.29–3.42), suggesting selective reporting of more extreme diagnostic contrasts in smaller cohorts (Supplementary Figures S10–S12).

## Discussion

### AI’s diagnostic performance compared to conventional methods

This meta-analysis demonstrated that AI-based imaging interpretation achieves a pooled AUC of 0.934 (partial AUC 0.81), with sensitivity and specificity of approximately 0.83 and 0.91, respectively. These results indicate AI’s substantial advantage over manual readings, quantified by a logit AUC mean difference of 0.43. Such high diagnostic accuracy suggests that AI tools could detect pulmonary hypertension (PH) earlier in the disease course, even when clinical signs are subtle. Timely detection is critical because delayed diagnosis of pulmonary arterial hypertension has been consistently linked to poorer patient outcomes, whereas early diagnosis allows patients to begin therapy earlier, improving their long-term prognosis (Ono et al., 2024).

Our findings align with established diagnostic frameworks outlined in the ATS/ERS and ESC/ERS guidelines, which advocate a tiered approach to pulmonary hypertension (PH) diagnosis, beginning with symptom evaluation and chest imaging, followed by echocardiography and definitive confirmation via right-heart catheterization (American College of Cardiology, 2022). As outlined by the American Thoracic Society (ATS), chest radiographs may show pulmonary artery enlargement or right heart changes suggestive of PH, though their diagnostic sensitivity remains limited (American Thoracic Society, 2023). Accordingly, the American College of Radiology (ACR) designates both chest X-ray and contrast-enhanced chest CT as appropriate first-line investigations in suspected cases (American College of Radiology, 2024). However, guidelines also emphasize that a normal chest X-ray does not exclude PH, underscoring the historical limitations of conventional interpretation (PHA Europe, 2022). Our meta-analysis suggests that AI integration can substantially mitigate this constraint. With a pooled AUC of 0.934, AI-assisted CXR interpretation demonstrated enhanced sensitivity in detecting subtle radiographic abnormalities that may be overlooked by human readers (Rajaram et al., 2015). These findings support the potential utility of AI as a triage tool, enabling earlier identification of high-risk individuals for echocardiographic evaluation while minimizing unnecessary invasive procedures in low-risk patients. Prior studies further corroborate AI’s diagnostic advantage over conventional radiologist interpretation in CXR-based PH screening (Rajaram et al., 2015). Thus, AI-enhanced chest radiography could reinforce and streamline the early diagnostic phase of PH, improving adherence to guideline-recommended diagnostic workflows (American College of Cardiology, 2022).

While chest radiography was the primary imaging modality in our meta-analysis (7 of 12 studies, 6,526 patients), current ESC/ERS guidelines endorse a multimodal approach to PH diagnosis, integrating echocardiography, CT, and MRI for complementary insights (American College of Cardiology, 2022). Echocardiography remains the cornerstone for noninvasive screening, with CT used to identify lung pathology and chronic thromboembolic PH (CTEPH), and MRI for advanced right ventricular assessment. AI can augment all of these modalities, quantifying pulmonary artery dimensions on CT, standardizing TR-jet velocity on echo, and improving ventricular measurements on MRI. Although fewer studies in our meta-analysis evaluated CT, echo, or CMR, the positive findings suggest AI’s value is not limited to CXR. As such, AI may support each step of imaging including broad CXR screening, detailed echo/CT evaluation, and precise MRI phenotyping, in line with current recommendations (American College of Radiology, 2024).

The meta-analysis further concluded that artificial intelligence (AI) outperformed conventional methods consistently across the area under the curve (AUC), a key parameter for measuring the accuracy of diagnosis, on multiple occasions. Across 12 studies involving 7,459 patients, Logit mean difference (MD) in AUC was 0.43 (95% confidence interval: 0.23–0.64; *p* < 0.0001) and reflects that AI can enhance diagnostic performance across imaging modalities. The low heterogeneity (*I*^2^ = 21.0%) further supports the consistency of this improvement across different studies and imaging modalities.

Complementing this, the bivariate random-effects meta-analysis revealed a pooled AUC of 0.91, underscoring the excellent overall diagnostic performance of AI-based models. When combined, these findings highlight that AI not only improves the average AUC compared to conventional methods but also achieves a high level of diagnostic accuracy across diverse imaging techniques and patient populations. The findings are in accordance with other studies that have concluded high AUC values in AI systems for ophthalmic and respiratory imaging, often superior to human experts in lesion detection and disease diagnosis tasks (Aggarwal et al., 2021; Najjar, 2023).

The logit MD in AUC between CXR and other modalities did not differ significantly, according to the imaging modality-based subgroup analysis. However, when examining the effect direction of AUC enhancement, a significant subgroup difference was found (*χ*^2^ = 5.30, df = 1, *p* = 0.0213). With no discernible heterogeneity (*I*^2^ = 0%, *τ*^2^ = 0, *p* = 0.7117), the group exhibiting an enhancing effect had a pooled logit AUC of 0.59 (95% CI: 0.35–0.83). Subgroup comparisons between various imaging modalities independently were also not statistically significant. The versatility of AI in imaging techniques is notably demonstrated by CMR, which was successful in a trial involving 220 patients. These results demonstrate AI’s versatility in imaging methods. A trial of AI-aided CT imaging for the diagnosis of COVID-19 showed improved diagnostic accuracy, pointing to the ability of AI to enhance CT-based diagnostics (Moezzi et al., 2021).

Further examination of diagnostic performance metrics showed that the pooled sensitivity was 0.824 (95% CI: 0.713–0.899), with a relatively low false positive rate of 0.097 (95% CI: 0.062–0.149). The summary receiver operating characteristic (SROC) curve visually confirmed this strong diagnostic accuracy, with the area under the curve reaching 0.934 and a partial AUC of 0.81, indicating that AI models maintain high sensitivity while controlling false positives effectively.

In addition to the bivariate analysis, univariate meta-analyses provided complementary insights: the pooled sensitivity was slightly higher at 0.83 (95% CI: 0.73–0.90), albeit with substantial heterogeneity (*I*^2^ = 83.8%), while the pooled specificity was 0.91 (95% CI: 0.86–0.95) with moderate heterogeneity (*I*^2^ = 41.5%). The diagnostic odds ratio (DOR), a composite measure of test effectiveness, was also notably high at 54.26 (95% CI: 22.50–130.87), supporting the strong discriminatory power of AI diagnostics despite some heterogeneity (*I*^2^ = 70.7%).

Taken together, these pooled sensitivity, specificity, and DOR values from both univariate and bivariate analyses reinforce the conclusion that AI-based diagnostic imaging offers very good to excellent diagnostic accuracy. The ROC curve analysis further substantiates this, illustrating that AI models achieve a robust balance between sensitivity and specificity, making them highly effective tools for clinical decision-making.

### Efficiency gains with AI integration

AI’s ability to process medical images rapidly is a key advantage, reducing diagnostic time and mitigating human error due to fatigue or oversight. For example, emergency settings benefit significantly from AI’s speed in analyzing complex data, enabling timely interventions. Additionally, AI enhances image quality through noise reduction and normalization techniques, improving visualization of anatomical structures critical for accurate diagnosis. Novak et al. (2024) found that AI-enhanced workflows in emergency radiology improved both efficiency and diagnostic accuracy.

### Challenges and methodological considerations

While its advantages are evident, there are challenges to implementing AI in diagnostic imaging:

*Overdiagnosis risks*: Oversensitivity can result in false positives or identification of clinically insignificant abnormalities, requiring stringent calibration of algorithms.

*Heterogeneity across studies*: Methodological and outcome measure differences make direct comparison between studies challenging and potentially exaggerate AI effectiveness.

*Bias risks*: Retrospective analysis and blinding in some but not all studies introduce bias in results, as indicated by QUADAS-2 evaluations with issues in patient selection and index tests.

Although 8 of the included studies were rated low risk for bias, 2 had some concerns, and 2 were rated as high risk, particularly in patient selection and flow/timing domains. Standardized reporting guidelines are necessary to maintain consistency and reliability in assessing AI’s performance in diagnosis. Robust validation and transparent reporting are stressed in literature concerning the state of AI in diagnostic imaging (Kusunose et al., 2022). In addition, the current landscape of AI adoption in diagnostic imaging is hindered by inconsistent regulatory frameworks, integration challenges, and a lack of clinician-centered design, factors that must be addressed to maximize clinical utility and trust (Larson et al., 2021).

The translation of the diagnostic performance of AI into real-world practice is conditioned by substantial deployment barriers. It relies heavily on robust computational infrastructure, smooth system connections, and seamless integration with PACS and EHR systems, which many under-resourced hospitals lack (Nair et al., 2022). Successful deployment in real-life situations requires careful planning, investment, and teamwork, especially in areas with limited support. Beyond technical deployment, the implementation of diagnostic AI in PH imaging raises important ethical and practical concerns. Although the overall FPR is less than 0.10, AI implementation still carries misdiagnosis risks from low false-positive/negative rates, which can trigger invasive tests, delay diagnosis, and cause anxiety (Bernstein et al., 2023). Moreover, algorithmic bias arises when models train on limited population samples, reducing generalizability and necessitating accountability through transparent documentation. Cybersecurity risks from large-scale data transfers further heighten practical concerns, demanding rigorous oversight by skilled professionals (Herington et al., 2023). Lastly, AI should act as a safety partner with humans, improving patient safety by providing an extra “pair of eyes” and detecting subtle pH signs while clinicians filter out clearly incorrect AI outputs and retain final decision-making authority. This setup reduces both missed diagnoses and unnecessary investigations (Cabitza et al., 2021).

### Clinical implications and future directions

The findings strongly favor the implementation of AI in standard diagnostic imaging practice based on its steady improvements in accuracy among modalities. Real-world application, though, will need to overcome limitations like overdiagnosis and methodologic heterogeneity while emphasizing clinically relevant endpoints such as patient survival and treatment response. Prospective studies assessing AI’s long-term advantages in various clinical settings are among the research priorities for future studies. Additionally, it is essential that explainable AI models are developed to increase clinician trust and enable the embedding of AI tools into clinical workflow.

Furthermore, leave-one-out sensitivity analysis confirmed the robustness of pooled estimates, and no significant small-study effects were detected using Egger’s test, supporting the reliability of the meta-analytic outcomes. However, several limitations related to heterogeneity exploration should be noted. Although we performed subgroup analyses by imaging modality, additional subgrouping by study design and by neural network architecture was not feasible because all included studies were observational, and each architecture was represented by only one study. Furthermore, the relatively small number of studies in individual comparisons precluded reliable meta-regression. In summary, artificial intelligence is an exciting evolution of diagnostic imaging with increased precision and decreased inefficiencies in inherent manual methods. Incorporation of this technology in health systems may potentially transform clinical decision-making as well as outcomes of patients greatly.

## Conclusion

Our meta-analysis provides well-supported evidence that artificial intelligence enhances diagnostic performance across key imaging modalities, including Chest X-ray, CT, and Echocardiography. The consistent improvement in AUC values across diverse study settings and patient populations emphasizes the potential of AI-assisted diagnostic interpretation. As diagnostic imaging continues to evolve, these findings support the integration of AI into routine practice, with the potential to boost accuracy, and enhance clinical decision-making. Future studies should focus on implementing these findings in real-world settings to ensure long-term benefits for patients.

## Data Availability

The original contributions presented in the study are included in the article/Supplementary material, further inquiries can be directed to the corresponding author.

## Glossary

AI: Artificial intelligence
AUC: Area under the (receiver-operating-characteristic) curve
CIs: Confidence intervals
CMR: Cardiac magnetic resonance imaging
CT: Computed tomography
CXR: Chest X-ray
DOR: Diagnostic odds ratio
FN: False negative
FP: False positive
FPR: False-positive rate
HSROC: Hierarchical summary ROC (if you keep that term)
***I*** ^2^: Higgins heterogeneity statistic
MRI: Magnetic resonance imaging
PH: Pulmonary hypertension
PRISMA-DTA: Preferred Reporting Items for Systematic Reviews and Meta-Analyses—Diagnostic Test Accuracy
QUADAS-2: Quality Assessment of Diagnostic Accuracy Studies-2
REML: Restricted maximum likelihood
RHC: Right heart catheterization
ROC: Receiver operating characteristic
SROC: Summary ROC
SN: Sensitivity
SP: Specificity
TP: True positive
PACS: Picture archiving and communication system
EHR: Electronic health record
