# Supplementary material for: Comparative accuracy of artificial intelligence versus manual interpretation in detecting pulmonary hypertension across chest imaging modalities: a diagnostic test accuracy meta-analysis
Source: Front Artif Intell. 2026 Jan 13;8:1709489. doi: 10.3389/frai.2025.1709489 (PMC12835279; doi:10.3389/frai.2025.1709489)
Supplement: Supplementary file 1 [file Data_Sheet_1.docx]

**Supplementary Material**

**Table S1: Search Strategy**

| Database | Search String |
| --- | --- |
| Pubmed | (("AI algorithm*"[tw] OR "artificial intelligenc*"[tw] OR "machine learning*"[tw] or "Deep Learning*"[tw] OR "Natural Language Processing*"[tw] OR "Machine Learning"[Mesh] OR "Artificial Intelligence"[Mesh] OR "Artificial Intelligence"[Mesh] OR "Natural Language Processing"[Mesh]) AND ("Pulmonary hypertension"[tw] OR "Pulmonary arterial hypertension"[tw] OR "Primary pulmonary hypertension"[tw] OR "Secondary pulmonary hypertension"[tw] OR "Pulmonary vascular disease*"[tw] OR "Hypertension, Pulmonary"[Mesh] OR "Pulmonary Arterial Hypertension"[Mesh])) AND ("Chest imaging*"[tw] OR "Chest X-ray*"[tw] OR "chest radiography*"[tw] OR "chest CT scan*"[tw] OR "thoracic imaging*"[tw] OR "chest ultrasound*"[tw] OR "lung imaging*"[tw] OR "heart imaging*"[tw] OR "chest MRI"[tw] OR "heart MRI"[tw] OR echocardiography[tw] OR electrocardiography[tw] OR "right heart catheterization"[tw] OR "Radiography, Thoracic"[Mesh] OR "Echocardiography"[Mesh]) |
| Embase | ('AI algorithm' OR 'artificial intelligence' OR 'machine learning' or 'Deep Learning' OR 'Natural Language Processing') AND ('Pulmonary hypertension' OR 'Pulmonary arterial hypertension' OR 'Primary pulmonary hypertension' OR 'Secondary pulmonary hypertension' OR 'Pulmonary vascular disease') AND ('Chest imaging' OR 'Chest X-ray' OR 'chest radiography' OR 'thoracic radiography' OR 'chest CT scan' OR 'thoracic imaging' OR 'chest ultrasound' OR 'lung imaging' OR 'heart imaging' OR 'chest MRI' OR 'heart MRI' OR 'echocardiography' OR 'electrocardiography' OR 'right heart catheterization') |
| Science Direct | ('artificial intelligence' OR 'Deep Learning' OR 'machine learning') AND ('Pulmonary hypertension' OR 'Pulmonary arterial hypertension') AND ('Chest imaging' OR 'echocardiography' OR 'electrocardiography' OR 'right heart catheterization') |
| Scopus | ('artificial intelligence' OR 'Deep Learning' OR 'machine learning') AND ('Pulmonary hypertension' OR 'Pulmonary arterial hypertension') AND ('Chest imaging' OR 'echocardiography' OR 'electrocardiography' OR 'right heart catheterization') |
| Cochrane | ID Search Hits  #1 (AI algorithm):ti,ab,kw 323  #2 (artificial intelligence):ti,ab,kw 2578  #3 (machine learning):ti,ab,kw 3266  #4 (Deep Learning):ti,ab,kw 1708  #5 (Natural Language Processing):ti,ab,kw 323  #6 MeSH descriptor: [Artificial Intelligence] explode all trees 3388  #7 MeSH descriptor: [Machine Learning] explode all trees 1082  #8 MeSH descriptor: [Deep Learning] explode all trees 356  #9 MeSH descriptor: [Natural Language Processing] explode all trees 76  #10 #1 OR #2 OR #3 OR #4 OR #5 OR #6 OR #7 OR #8 OR #9 8477  #11 (Pulmonary hypertension):ti,ab,kw 6427  #12 (Pulmonary arterial hypertension):ti,ab,kw 2892  #13 (Pulmonary vascular disease):ti,ab,kw 2013  #14 MeSH descriptor: [Hypertension, Pulmonary] explode all trees 1682  #15 MeSH descriptor: [Pulmonary Arterial Hypertension] explode all trees 208  #16 #11 OR #12 OR #13 OR #14 OR #15 7566  #17 (chest imaging):ti,ab,kw 2887  #18 (chest radiography):ti,ab,kw 1940  #19 (chest MRI):ti,ab,kw 696  #20 (echocardiography):ti,ab,kw 15104  #21 (electrocardiography):ti,ab,kw 15612  #22 (right heart catheterization):ti,ab,kw 1119  #23 (Chest X-ray):ti,ab,kw 3953  #24 (chest CT scan):ti,ab,kw 1154  #25 MeSH descriptor: [Radiography, Thoracic] explode all trees 494  #26 MeSH descriptor: [Echocardiography] explode all trees 5222  #27 MeSH descriptor: [Electrocardiography] explode all trees 10602  #28 #17 OR #18 OR #19 OR #20 OR #21 OR #22 OR #23 OR #24 OR #25 OR #26 OR #27 36993  #29 #10 AND #16 AND #28 5 |

**Figure S1: The Risk of Bias using the QUADAS-2 tool**


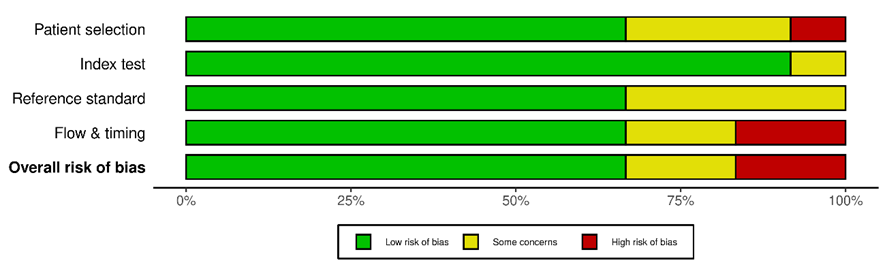

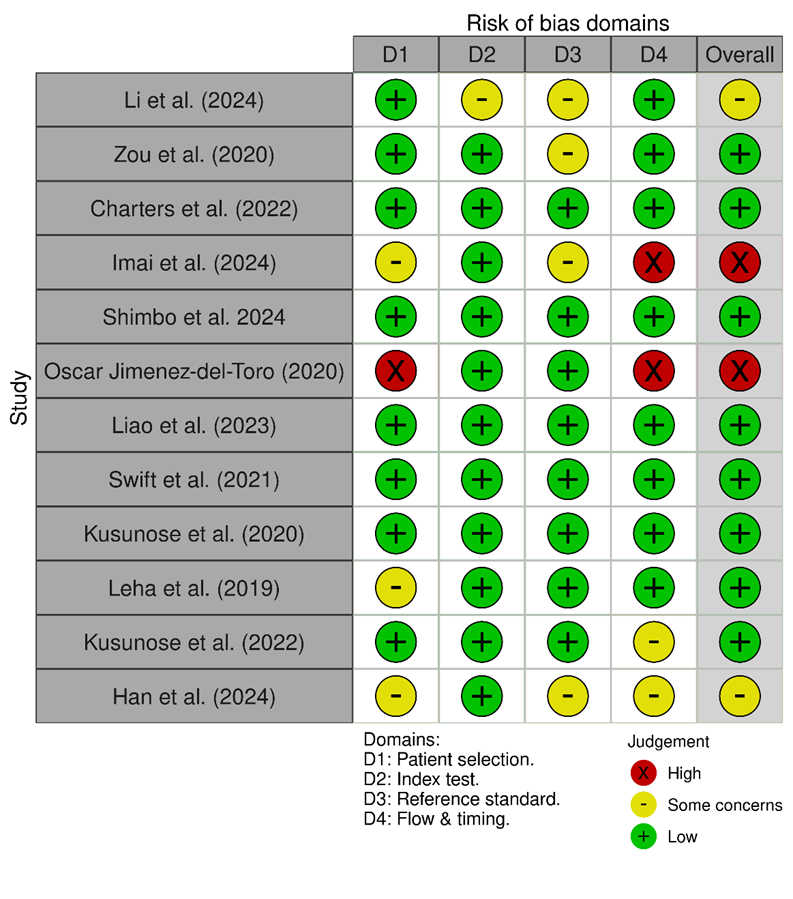


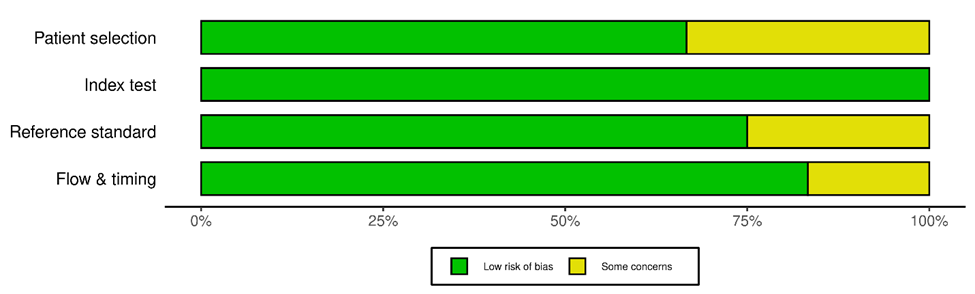

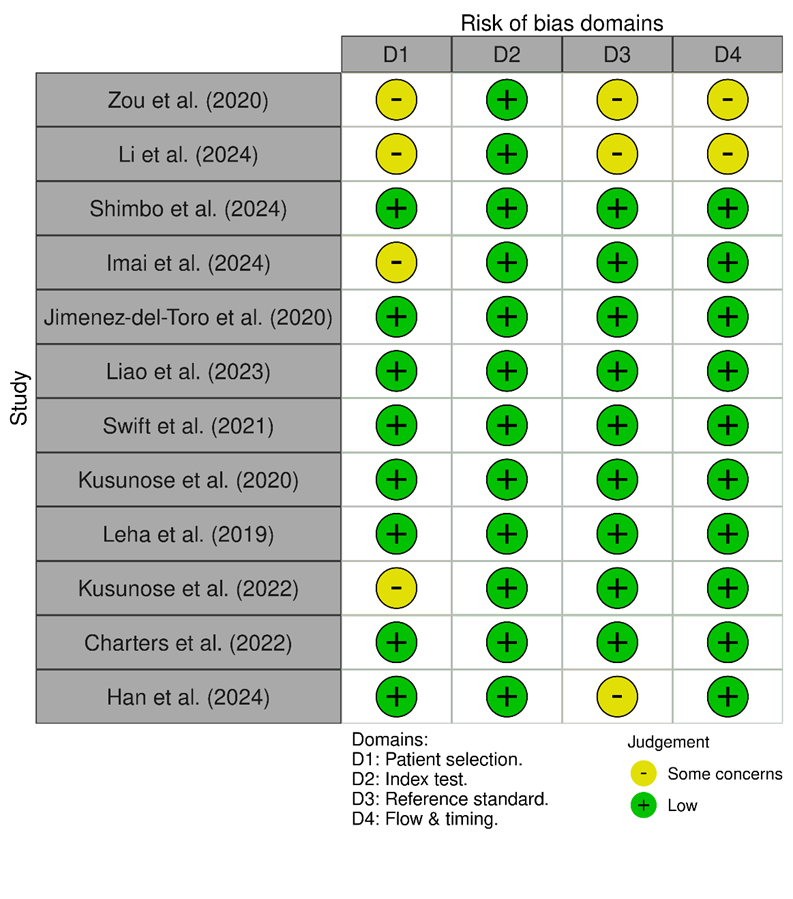


**Figure S2: Forest plot of Sensitivity analysis of Logit-Transformed Mean Difference in AUC**

**
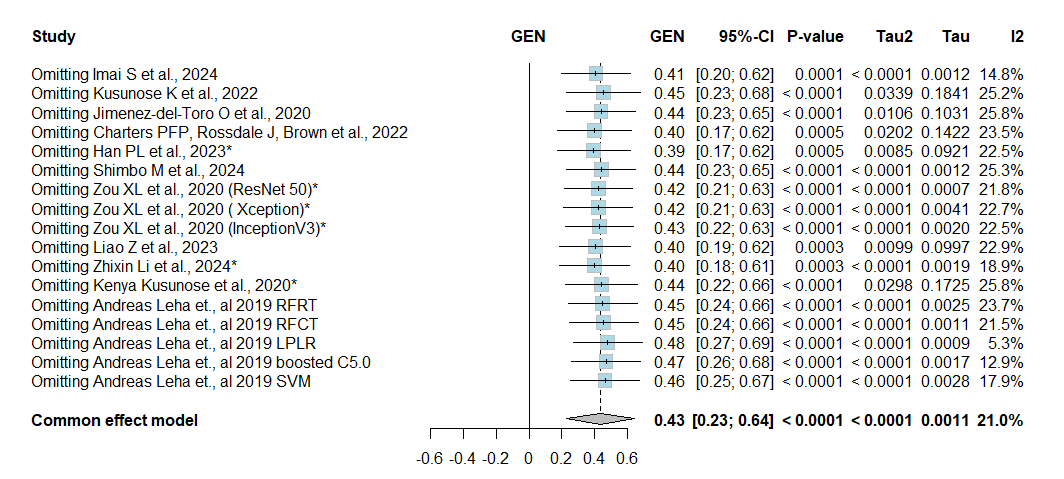
**

**Figure S3: Logit-Transformed Mean Difference in AUC: Subgroup analysis based on Chest X-ray vs other imaging modalities**

**
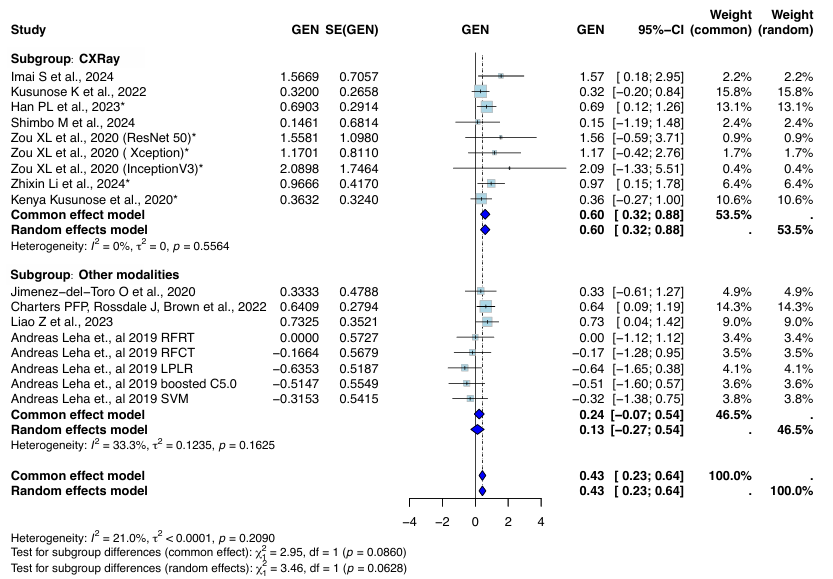
**

**Figure S4: Logit-Transformed Mean Difference in AUC: Subgroup analysis based on X-ray vs CT vs echocardiography**

**
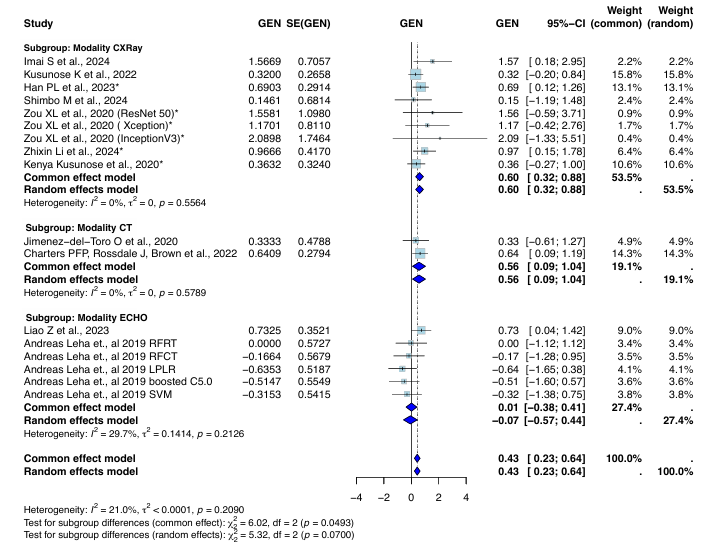
**

**Figure S5: Forest Plot of Sensitivity (Univariate Analysis)**

**
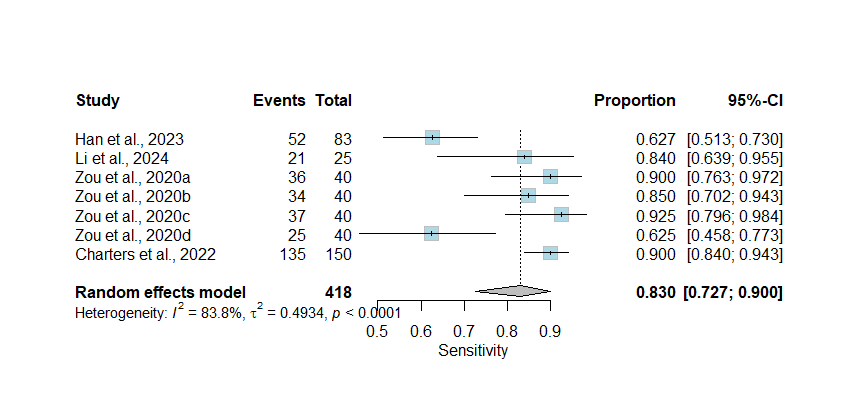
**

**Figure S6: Forest Plot of Specificity (Univariate Analysis)**

**
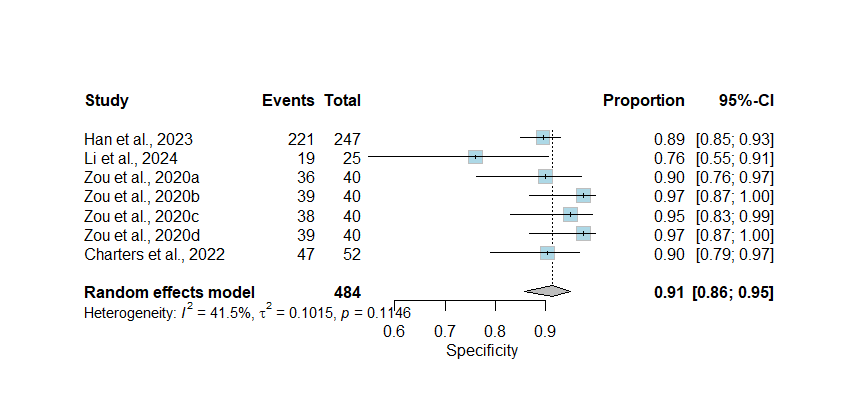
**

**Figure S7: Forest Plot of Diagnostic Odds ratio (Univariate Analysis)**

**
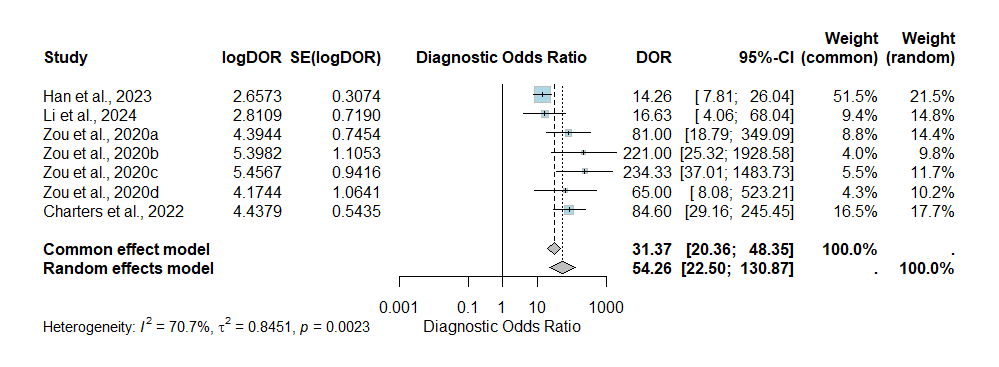
**

**Figure S8: Forest Plot of Leave-one-out analyses of Sensitivity (Univariate Analysis)**

**
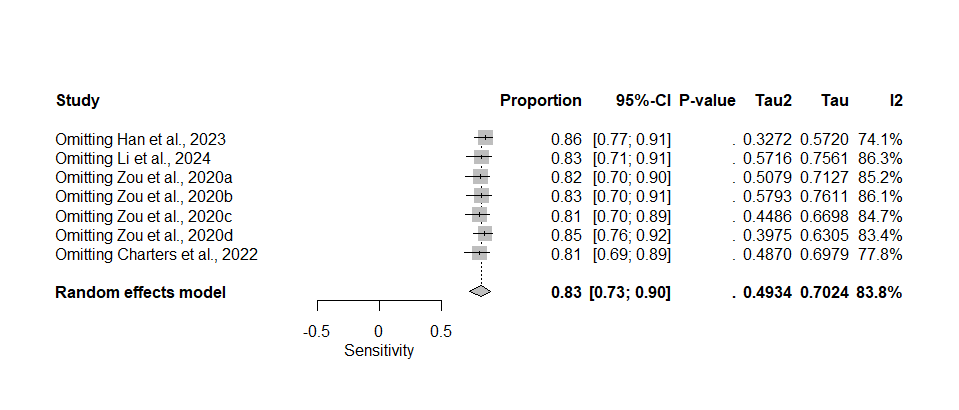
**

**Figure S9: Forest Plot of Leave-one-out analyses of Diagnostic odds ratio (Univariate Analysis)**

**
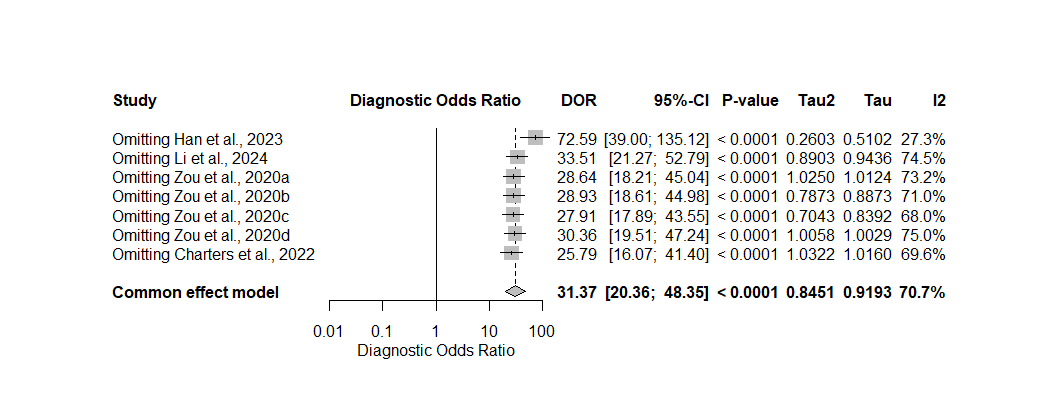
**

**Figure S10: Funnel Plot of Sensitivity (Univariate Analysis)**

**
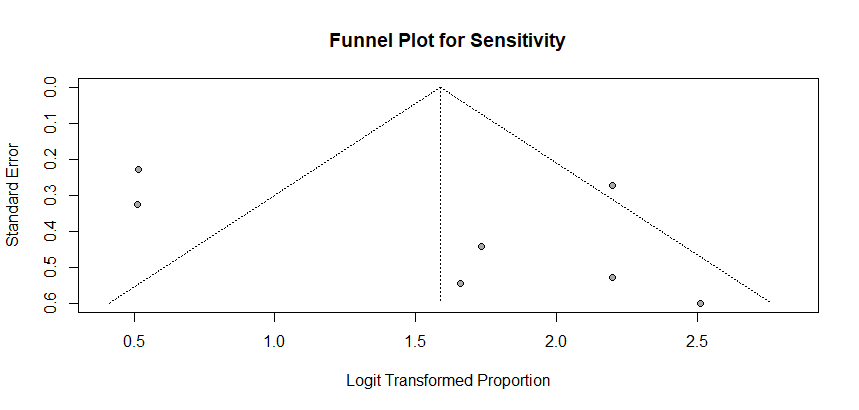
**

**Figure S11: Funnel Plot of Specificity (Univariate Analysis)**

**
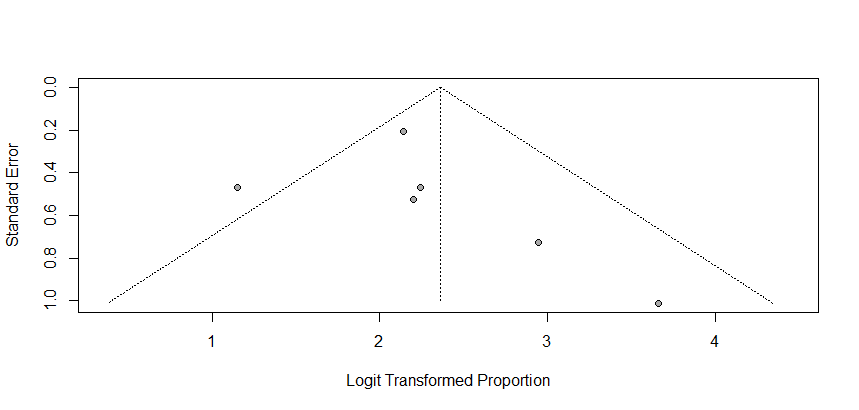
**

**Figure S12: Funnel Plot of Diagnostic odds ratio (Univariate Analysis)**

**
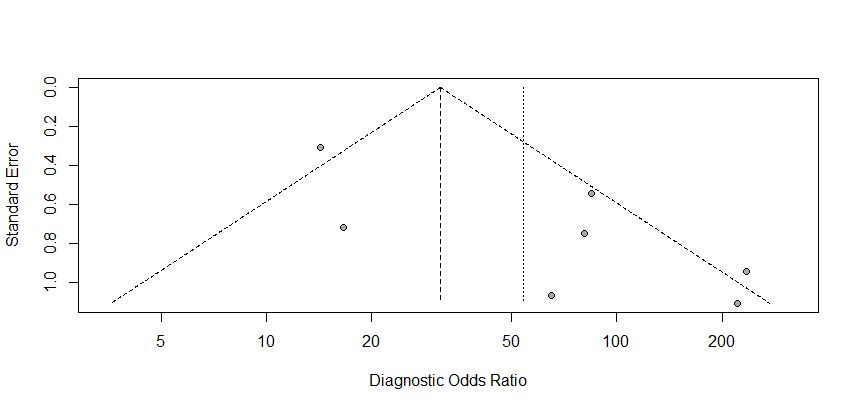
**
